# Greener Extraction Solutions for Microalgal Compounds

**DOI:** 10.3390/md23070269

**Published:** 2025-06-27

**Authors:** Gwendoline Kopp, Chiara Lauritano

**Affiliations:** 1 Marine Biology Research Group, Ghent University, Krijgslaan 281, B-9000 Gent, Belgium; gwendoline.kopp@imbrsea.eu; 2 Ecosustainable Marine Biotechnology Department, Stazione Zoologica Anton Dohrn, Via Ammiraglio Ferdinando Acton 55, 80133 Napoli, Italy

**Keywords:** microalgae, green extraction, bioactive compounds, lipids, pigments

## Abstract

Conventional methods for extracting bioactive compounds from microalgae rely on organic solvents that are both polluting and potentially harmful to human health. In recent years, a noticeable shift has emerged toward greener extraction alternatives that are more environmentally friendly and sustainable. This review highlights various green extraction techniques, compounds, and yields obtained from different microalgal species for a range of applications and provides a comparison between the yields of conventional and green extraction methods. Green extraction methods have shown yields that are comparable to, or even exceed, those of conventional techniques, although they are predominantly studied for the extraction of lipids and pigments. This review aims to provide an overview of the current state of green extraction applied to microalgae, and to outline future research perspectives in this emerging field.

## 1. Introduction

Microalgae represent the most diverse group of photosynthetic eukaryotic organisms, thriving across a wide range of ecosystems, including freshwater, brackish, and marine environments [1]. Their cultivation is relatively simple compared to marine plants and other macroorganisms, and they exhibit rapid growth rates [2]. Microalgae are characterized by their remarkable biodiversity and their ability to grow under extreme environmental conditions, such as variations in temperature, salinity, pH, and light intensity [2]. This adaptability makes them excellent producers of high-value compounds [3]. Among microalgae, diatoms are a particularly notable group, comprising over 100,000 species [3]. As a major component of phytoplankton, diatoms contribute to approximately 50% of global oceanic primary production [4]. The diverse applications of diatoms span sectors such as biofuels, nutrition, biomolecular materials, and nanotechnology [5]. In addition, other groups of microalgae, such as green algae, flagellates, and dinoflagellates, are being explored for their biotechnological potential [6,7,8,9].

Microalgae have recently gained attention as potential sources of bioactive compounds derived from their primary and secondary metabolism [10]. These metabolites present promising applications across various sectors, including nutrition, biofuels, cosmetics, and pharmaceuticals [3,10].

The nutritional sector represents the foremost area for the commercialization of microalgae, given their richness in proteins, polyunsaturated fatty acids (PUFAs), pigments, vitamins, and minerals [11]. Microalgae can be consumed either in their raw form or as processed products [12]. Notably, species such as *Limnospira* (formerly *Spirulina* or *Arthrospira*) and *Chlorella* exemplify the nutritional potential of microalgae, contributing to a global market worth hundreds of millions of dollars [13]. Microalgae are most consumed in their raw form as dietary supplements, either in capsule form (e.g., Dunaliella by Terra&Vita, Organic Chlorella Tablets by Bulk, or Spirulina by Nature’s Way) or as powders (e.g., Chlorelle Poudre by Sol Semilla, Spirulina Powder by Bulk, Polvere di Alga Kelp Bio, or a kelp powder by ErbaVoglio).

Over the past two decades, the use of microalgae as a biodiesel source has gained significant interest [14]. Unlike terrestrial crops, which pose challenges such as inefficient land use, low lipid yield, and high water consumption, microalgae offer a more sustainable alternative [15]. Certain microalgal strains are capable of accumulating substantial lipid content within their cells, which can be converted into biodiesel [16] by thermochemical or biochemical conversion [17]. The microalgae *Chlorella*, *Nannochloropsis*, and *Phaeodactylum* contain between 20–50% lipids (as a % of dry weight biomass) and exhibit significantly higher productivity compared to lipid-rich species such as *Botryococcus braunii* [18]. However, to fully replace petroleum-derived liquid fuels with oil extracted from microalgae, advancements are required in algal biology through genetic and metabolic engineering, as well as in photobioreactor technology [16]. Photobioreactors are a critical tool for optimizing algal cultivation and enhancing biofuel production [19].

Numerous studies have demonstrated the antioxidant potential of bioactive compounds such as pigments, polyphenols, and vitamins derived from microalgae, making them a promising source for novel bioproducts, particularly in the cosmetic industry [20]. Due to their exposure to environmental stresses, such as UV radiation and high salinity, microalgae produce active compounds like chlorophyll and carotenoids, which are potential candidates for developing bioproducts to protect the skin from sun-induced damage [20]. For example, *Chlorella* microalgae extract has been shown to stimulate collagen synthesis, providing essential support for skin tissue and regeneration [21]. Additionally, compounds such as polysaccharides, hydroxy acids, and exopolysaccharides extracted from microalgae can act as effective moisturizers [20]. While the use of these bioactive compounds as active ingredients in cosmetics is becoming more widespread, their applications in the industry often remain limited to roles such as thickening, gelling, and antioxidant agents [22,23]. Various bioactive compounds derived from microalgae are found in cosmetic products, such as phycocyanin from *Arthrospira platensis* (Spiruderm^®^ by AlgoSource), and exopolysaccharides (EPS) from *Porphyridium cruentum* (Exopolysaccharides–EPS de *Porphyridium* by AlgoSource). Additionally, Yves Rocher France offers a range of six products under the Pure Algue line by Cosmétique Végétale^®^, which utilizes raw extracts of *Tetraselmis*. Expanding the utilization of microalgae-derived compounds in cosmetics offers an opportunity to develop multifunctional and sustainable skincare products.

The use of microalgae in the pharmaceutical sector is still in its early stages but shows significant promise, particularly as most microalgae are recognized under the Generally Recognized as Safe (GRAS) status [24]. Species such as *Dunaliella*, *Chlorella*, and *Spirulina* are notable for their production of polyunsaturated fatty acids (PUFAs), which have demonstrated efficacy in the prevention or treatment of cardiovascular diseases [25]. For example, *Arthrospira*, due to its high γ-linolenic acid (GLA) content, reduces lipid levels in patients with hyperlipidemic nephrotic syndrome, thereby potentially contributing to an indirect reduction in cardiovascular risk [26]. Carotenoids and sterols from microalgae also demonstrated an ability in the prevention of cardiovascular diseases [27]. PUFAs derivatives, eicosapentaenoic acid (EPA), docosapentaenoic acid (DPA), and (GLA) also show potential in managing type 2 diabetes, skin disorders, and asthma [25]. The following bioactive compounds—phenols, flavonoids, and sterols—extracted from *Nannochloropsis* sp. have demonstrated antidiabetic properties for type 2 diabetes by restricting α-glucosidase and α-amylase enzyme [28]. Pigments recovered from *Porphyridium* sp. showed potential for α-glucosidase inhibition and, hence, for antidiabetic activities [29]. The pigments potentially responsible for this inhibition include phycoerythrins, phycocyanins and allophycocyanins, which have been shown to exhibit enhanced production in media containing Fe^3+^ and Co^2+^, highlighting the need to optimize culture parameters [29]. Furthermore, polysaccharides derived from *Chlorella vulgaris* have exhibited significant antiviral activity [30] and immunomodulatory effects [31]. Triglycerides rich fraction of *Skeletonema costatum* and nucleosides rich fraction of *S. dohrnii* exhibited immunostimulant activity on human peripheral blood mononuclear cells (PBMCs) [32]. Bioactive compounds from microalgae are also being explored as potential anti-Alzheimer’s disease agents by inhibiting glutaminyl cyclase (QC), a key enzyme involved in the disease’s onset [33], astaxanthin microencapsulated in *Spirulina* has demonstrated neuroprotective benefits in rats [34]. Microalgae also possess antiproliferative properties, inducing cell cycle arrest and apoptosis, positioning them as promising candidates for anticancer drug development [35]. Cancer, which is not a single disease but rather a conglomerate of over 100 different types, remains one of the leading causes of mortality worldwide, claiming millions of lives annually [36]. Recent studies have highlighted the anticancer potential of *Skeletonema marinoi*, a eukaryotic photosynthetic diatom, with effects on lung cancer A549 and colon cancer COLO 205 tumor cells [3]. Similarly, an investigation of 32 microalgae species, including diatoms, dinoflagellates, and flagellates, identified diatoms as the only group exhibiting bioactivity, underscoring their capacity to produce bioactive compounds for therapeutic applications [3]. These findings reinforce the potential of microalgae as a rich source of bioactive compounds for pharmaceutical applications, particularly in the development of novel treatments for cancer and other diseases.

All the listed sectors above, among many others, rely on the extraction of bioactive compounds from microalgae. Currently, conventional extraction methods remain predominant. However, these traditional methods are often designed to isolate only a single class of molecules [37], which limits the diversity of compounds extracted and consequently reduces their potential applications. Conventional extraction methods have several drawbacks, including being time-consuming, labor-intensive, and exhibiting low selectivity and/or extraction yields [38]. Additionally, they typically require large quantities of organic solvents [38], and thus many hazardous residues [39]. In 2014, a total of 25.45 billion pounds of toxic chemical waste related to production activities was reported in the United States according to the Toxics Release Inventory [40]. These methods, such as Soxhlet extraction, Folch extraction, the Bligh and Dyer method, and maceration, not only pose significant environmental and health risks but also have considerable limitations in terms of sustainability [37]. The Folch and Bligh and Dyer methods are commonly used for lipid extraction, with Bligh and Dyer being less exhaustive, and therefore one of the most recommended methods for determining total lipid content [37,41]. Furthermore, at an industrial scale, conventional extraction processes can consume up to 50% of the total energy required for the entire production chain, further highlighting their low efficiency and environmental impact [42].

New extraction techniques are emerging that require little to no toxic solvents, thereby minimizing environmental impact [37,42]. These methods can not only reduce extraction time but also enhance the yield of bioactive compounds [39]. Among various industries, the cosmetic sector has been at the forefront of adopting greener alternatives. The solvents used in this field must comply with strict regulatory standards, leading to the gradual phase-out of hazardous solvents such as chloroform and phenol, which were previously employed for extract enrichment or metabolite isolation. These are now being replaced by green solvents, such as ethanol and water, particularly in cosmetics [43]. Green extraction, as defined by Chemat et al. [42] (p. 2), refers to “the discovery and design of extraction processes that reduce energy consumption, allow the use of alternative solvents and renewable natural products, and ensure a safe and high-quality extract/product” and contains six principles: (i) innovation by selection of varieties and use of renewable plant resources; (ii) use of alternative solvents and principally water or agro-solvents; (iii) reduce energy consumption by energy recovery and using innovative technologies; (iv) production of co-products instead of waste to include the bio- and agro-refining industry; (v) reduce unit operations and favor safe, robust, and controlled processes; and (vi) aim for a non-denatured and biodegradable extract without contaminants [42].

Previous reviews in the field considered only some of the techniques reported here, or only one technique, or they did not focus on the extracted compounds [37,39,42]. For this review, the keywords “green extraction” and “microalgae” were used to search publications on PubMed and Google Scholar. All publications from our literature research mentioning green techniques were included, without any time restriction. The current review focuses on green extraction techniques used for microalgae, comparing their efficiency and bioactive compound yields with those obtained through conventional extraction methods and critically discussing their strengths, weaknesses, opportunities, and threats.

## 2. Green Extraction Techniques

### 2.1. Supercritical Fluid Extraction (SFE)

Supercritical fluid extraction (SFE) using carbon dioxide (CO_2_) is a non-conventional extraction method that has demonstrated its efficiency in recovering high-value compounds from microalgae [44] since the 1970s [42]. SFE can enable higher extraction selectivity and shorter processing times at optimized conditions compared to conventional extraction methods [45]. Indeed, according to Nobre et al. [46], the extraction of lipids from *Nannochloropsis* sp. using Soxhlet reached 40.7% lipid recovery and 25.3% for the Bligh and Dyer method against 45% of lipid recovery using SFE-CO_2_ with the addition of ethanol as a co-solvent. The thermodynamic properties and heat transfer characteristics of CO_2_ make it the most widely used solvent for SFE. Additionally, the extraction of thermolabile molecules via SFE-CO_2_ prevents degradation or changes in the chemical composition of metabolites [45,47]. Moreover, CO_2_ is non-flammable, has low toxicity, is cost-effective compared to organic solvents [48], and is recognized as a GRAS solvent [49]. However, a major limitation of this technique is the non-polarity of CO_2_, which restricts its ability to extract polar compounds. To overcome this limitation, a co-solvent with opposite polarity, such as water, ethanol, or methanol, can be added [45]. Another limitation is the high initial investment cost, estimated at approximately USD 170,000 for an extraction vessel with a 500 mL capacity (Waters, Milford, MA, USA) [39]. Nevertheless, SFE is economically advantageous due to its closed-system design, allowing for CO_2_ and co-solvent recovery while preventing solvent residues in the final extract [45]. This technique is widely applied in various industries, including food, cosmetics, and pharmaceuticals [42].

SFE is based on the use of an extraction fluid maintained at supercritical conditions by applying pressure and temperature above its critical point (for CO_2_: 31.1 °C, 73.8 bar) [45,49]. The process typically begins with biomass pretreatment, either chemically or mechanically, to enhance metabolite recovery, such as fatty acids [50]. According to Esquivel-Hernández et al. [39] (p. 217) the general steps of the SFE process are as follows: (i) the biomass is loaded and absorbs the supercritical solvent; (ii) soluble compounds dissolve into the solvent; (iii) the dissolved compounds diffuse to the solid surface; (iv) the solvent transports the compounds and are removed from the extractor. Operating conditions, such as CO_2_ flow rate (g/min), extraction time (min), temperature (°C), and pressure (bar), vary depending on the target metabolites and biomass type [51]. Temperature and pressure play a crucial role in the SFE-CO_2_ process due to their combined effect on solvent density and solute vapor pressure [52].

SFE-CO_2_ enables the extraction of a wide range of bioactive compounds from freshwater and marine microalgae, generally of low molecular weight [42]. These include fatty acids such as saturated fatty acids (SFAs), monounsaturated fatty acids (MUFAs), polyunsaturated fatty acids (PUFAs), including eicosapentaenoic acid (EPA) docosahexaenoic acid (DHA), and γ-linolenic acid (GLA), diolefins, carotenoids such as β-carotene, fucoxanthin, and astaxanthin, phenolic compounds from various food waste and microalgae/cyanobacteria sources, terpenes (mono- and diterpenes), proteins, carbohydrates, and sterols [39,44,49,52,53,54,55].

### 2.2. Microwave-Assisted Extraction

Microwave-Assisted Extraction (MAE) is a non-conventional green extraction technique first introduced in 1986, which has gained significant interest in recent years due to its automation capability, short extraction times, and reduced use of organic solvents—requiring up to ten times less solvent compared to conventional methods. This reduction mitigates pollution, lowers production costs, and increases both the yield and quality of the extract [56,57,58,59]. According to Krishnan et al. [60], the extraction of lipids from *Chlorella vulgaris* using MAE, with a solution of ionic liquids as solvent, yielded 19.2% of lipids while with the conventional Bligh and Dyer method only 10.9% of lipids were yielded. MAE utilizes microwave energy to heat the solvent in contact with the sample of interest through ionic conduction and dipole rotation, facilitating the separation of bioactive compounds into the solvent phase [56,57]. The initial investment cost for a MAE vessel is lower than SFE or Pressurized Liquid Extraction (PLE), with an estimated cost of approximately USD 50,000 (CEM, Matthews, NC, USA) [39]. One of the main limitations of this technique is the dependency of microwave absorption on the solvent used, as heat generation by MAE varies according to the dielectric properties of the solvent [57]. Several solvent mechanisms can be employed in MAE: (i) a single solvent with strong microwave absorption, (ii) a combination of solvents with high and low dielectric losses (a measure of efficiency in converting microwave energy into heat), or (iii) a microwave-transparent solvent (which cannot be heated by microwaves) when extracting compounds from samples with high dielectric loss [57]. The extraction of microalgal compounds by MAE aligns with four of the six green extraction principles outlined by Chemat et al. and Bermúdez Menéndez et al. [42,61]. This technique is widely applied in the extraction of bioactive and nutraceutical compounds from microalgae, biodiesel recovery, and the isolation of compounds for the food industry [49,59,62].

According to Sparr Eskilsson and Björklund [57] (p. 232), the extraction process follows these steps: the sample is placed in the extraction vessel, and the solvent is added. Microwave radiation is then applied, initiating a pre-extraction phase to heat the solvent to the desired conditions. The sample is subsequently irradiated and extracted at the target temperature for a set duration (static extraction). Key parameters influencing MAE include the solvent/sample ratio, which directly affects the transfer of target metabolites (e.g., microalgal compounds) between the sample and the solvent. Temperature is another crucial factor, as it is directly influenced by microwave power, highlighting the importance of optimizing process parameters to minimize energy waste [39].

MAE enables the extraction of various bioactive compounds from freshwater and marine microalgae, particularly lipids such as essential oils and fatty acids (including PUFAs such as EPA and DHA), terpenoids (phytol and fucosterol), pigments (phycobiliproteins, phycoerythrin, carotenoids, and chlorophyll), phenolic compounds from soils, sediments, food by-products, and microalgae, as well as sulfated carbohydrates like fucoidans [39,49,57,63,64,65,66].

Although MAE is considered a green extraction technique, it still relies on small amounts of organic solvents such as hexane or chloroform. Future research should focus on the use of green solvents in MAE [39]. Additionally, while lipid extraction using MAE is well-documented, the extraction of other high-value compounds remains underexplored [67].

### 2.3. Pressurized Liquid Extraction (PLE)

Pressurized Liquid Extraction (PLE) is a more recent green extraction technique compared to SFE and MAE, emerging in 1995 [68]. In the literature, PLE is also referred to as Pressurized Solvent Extraction (PSE), Accelerated Solvent Extraction (ASE), or Enhanced Solvent Extraction (ESA) [39]. The principle of PLE involves applying high pressure (34 bar–204 bar) to maintain the solvent in its liquid state even at temperatures (20 °C–200 °C) above its boiling point [68,69]. PLE is characterized by short extraction times due to automation and the ability to process multiple samples simultaneously. A key advantage of this technique is its reduced consumption of organic solvents, as well as the possibility of using green solvents [70]. Additionally, the controlled solvent properties allow for higher extraction yields compared to conventional extraction [71]. A study from Cha et al. [71], showed promising results in the extraction of pigments from *Chlorella vulgaris* using PLE compared with Soxhlet and maceration extraction techniques. PLE with ethanol as a co-solvent yielded 3.78 mg/g of lutein and 9.63 mg/g of chlorophyll a, while Soxhlet yielded 3.42 mg/g of lutein and 3.32 mg/g of chlorophyll a, and 2.97 mg/g of lutein and 4.26 mg/g of chlorophyll a using maceration extraction. However, two main limitations of PLE include its tendency to produce non-selective extractions, highlighting the importance of solvent choice [72], and its high initial investment cost, estimated at approximately USD 140,000 (Thermo-Fisher, Waltham, MA, USA) [39]. PLE is primarily applied in the food, cosmetic, and pharmaceutical industries [68]. This technique is compatible with various solvents, making it a suitable option for green solvents such as limonene, which has been proposed as a replacement for hexane—a solvent with known environmental and toxicological concerns, particularly related to neuropathies [73]. Limonene has a dielectric constant similar to hexane [74] and is a by-product of the citrus fruit industry [75].

According to Waldebäck [68] (pp. 26–27) and Esquivel-Hernández et al. [39] (p. 223), the PLE process follows these steps: the matrix (e.g., microalgal dry biomass) is loaded into the extraction cell and filled with solvent. Temperature and pressure are then applied, with a pre-heating step necessary to desorb the target compounds from the matrix. This is followed by a static extraction phase, during which the compounds of interest diffuse into the matrix pores before being extracted. Multiple static extraction steps (one to five cycles) can be performed sequentially. After extraction, the cell is washed with fresh solvent, and pressurized nitrogen is injected to purge the solvent, allowing for the recovery of the extract. A crucial parameter in PLE is solvent selection, as extraction selectivity can only be achieved by adjusting the solvent composition or solvent mixtures according to the target compounds [68]. Additionally, temperature and extraction time are key factors influencing efficiency [76].

PLE enables the extraction of a wide range of bioactive compounds from freshwater and marine microalgae, including essential oils, pigments such as carotenoids (xanthophylls, β-carotene, lutein), chlorophyll a and b, phycobiliproteins, fatty acids such as saturated fatty acids, GLA, MUFAs, PUFAs such as EPA, polyphenols, and proteins [39,77,78,79,80,81,82,83,84].

### 2.4. Ultrasound-Assisted Extraction (UAE)

Ultrasound-Assisted Extraction (UAE) is a widely developed green extraction technique in the food industry [85], and is increasingly being adopted in other sectors. This method provides high purity of the final product and high extraction yields, making it more economical and eco-friendlier than conventional techniques. In the study of Zhao et al. [86], the use of UAE as a green extraction technique with cultivation media as a solvent yielded 36.85 g/100 g of carbohydrates while only 9.06 g/100 g of carbohydrates were yielded by solvent extraction from *Chlorella* sp. UAE significantly reduces solvent consumption—in some cases, no solvent was used—and shortens extraction time [81,85]. This technique appears to be well-suited for industrial-scale up, given its low investment cost and the possibility of modifying existing ultrasound extraction reactors already used in the food and chemical industries [85]. The principle of UAE relies on the propagation of ultrasound waves through the solvent, creating cavitation phenomena. This phenomenon disrupts cell walls, allowing the extraction of target compounds [87], depending on their solubility in the solvent [88]. Ultrasound application can also be used as a pretreatment, enhancing cell membrane permeability, either alone or in combination with other green extraction techniques, such as MAE [89].

According to Usman et al. [90] (p. 10) UAE process consists of the following steps: the biological matrix is suspended in a solvent, and a probe transmits ultrasound waves at a selected frequency (>20 kHz) and power for a defined extraction time. Several studies indicate that lower temperatures are preferable to prevent degradation of thermolabile bioactive compounds and improving yield recovery [88,89].

One of the limiting factors of this technique is the choice of solvent. The use of toxic solvents does not necessarily comply with green chemistry principles, and further research is needed to explore the application of UAE with green solvents [91]. However, commonly used solvents include acidified water, alcohols, acetone, hexane, and water [81,90]. While acetone is flammable and irritating, hexane has negative environmental and toxicological effects [83]. The selection of a solvent depends on the solubility of the target bioactive compounds as well as viscosity, surface tension, and vapor pressure of the solvent [88], physical parameters that can be affected by the applied temperature, and thereby the cavitation phenomena.

UAE technique enables the extraction of high-value compounds from marine and freshwater microalgae, including polyphenols, sterols, chlorophylls, carotenoids such as lutein, lipids such as PUFAs, MUFAs, and SFAs, and polysaccharides [81,85,91,92,93].

### 2.5. Subcritical Water Extraction (SWE)

Subcritical water extraction (SWE) is one of the green extraction techniques developed for the recovery of high-value bioactive compounds. SWE is applied in the food, pharmaceutical and bioenergy sectors for compounds extraction [94,95] and is well documented for plant extraction (excluding microalgae). This technique offers higher selectivity, shorter extraction times, and eliminates the need for toxic organic solvents, as it uses water as solvent [48]. Consequently, SWE has gained increasing attention due to its safe utilization, and eco-friendly nature [94]. In the literature, SWE can be found under different names, such as Pressurized Hot Water Extraction (PHWE) or SuperHeated Water Extraction (SHWE) [96]. This extraction technique uses water as a solvent and applies temperature and pressure, following a principle like PLE (Section 2.3). The water is maintained at a temperature between its boiling point and critical point (100 °C–374 °C) under pressure (10 bar–221 bar), ensuring it remains in a liquid state while enhancing the solubility of target compounds for extraction [76,94]. SWE offers several advantages but also has limitations. Due to the use of high temperatures, this technique may not be suitable for thermolabile compounds [94]. Additionally, subcritical water can induce oxidation of certain compounds. To mitigate this issue, water degassing through sonication or purging with dinitrogen (N_2_) can be implemented [94].

The SWE process consists of the following steps: (i) The matrix and solvent are either loaded into the extraction cell (static extraction) or pumped into the extraction cell (dynamic extraction). (ii) Extraction begins once the oven reaches the desired temperature and pressure is applied. (iii) After the extraction time, the extract is collected [48,76,94,95,96]. The metabolites are diffused to the surface of the matrix (e.g., microalgae), are dissolved in the water solvent and are eluted through the extraction cell [96]. The main factor influencing SWE is temperature. Subcritical water exhibits high diffusivity, low viscosity, and low surface tension, which reduces its dielectric constant and enhances the solubility of target bioactive compounds in water [76,94]. Therefore, it is essential to optimize the applied temperature based on the metabolites of interest. In contrast, pressure does not directly affect metabolite recovery but is necessary to maintain water in its liquid state during the extraction process [76].

Compounds from microalgae extracted by SWE are phenols, proteins, polysaccharides, amino acids such as glutamic acid, glucose, carbohydrates breakdown such as (di-_D_)-fructose anhydrides (DFAs) and lipids, and EPA from marine and freshwater microalgae [97,98,99,100,101,102].

### 2.6. Enzyme-Assisted Extraction (EAE)

The composition of the microalgal cell wall depends on the taxonomic group to which they belong. Consequently, the recovery of bioactive compounds is highly dependent on cell wall disruption. Techniques such as microwave or ultrasonic waves are commonly used for this purpose, but they involve high costs [103]. The use of enzymatic hydrolysis for cell wall disruption and subsequent extraction of bioactive compounds presents an economically attractive alternative, enabling high product yields and high-quality recovery [103,104] at laboratory scale [105]. The choice of enzyme or enzymes mixture—leading to higher yields [106]—depends on the enzyme’s specificity and region-selectivity towards the target compounds while preserving their properties [104,107]. EAE, when combined with other green extraction techniques, could further enhance the extraction efficiency of compounds from the microalgal matrix [108,109]. Depending on the composition of the microalgal cell wall and the target compounds, different enzymes can be used. Table 1 summarizes the various enzymes employed based on the microalgal species and the extracted compounds.

The EAE process follows these steps: a microalgal paste is mixed with a specific enzyme or enzyme mixture in a solution at an active pH, placed in a centrifuge tube, and incubated in a water bath or a thermostated system while being magnetically stirred for the required extraction time [103,106,110]. EAE offers several advantages, including operation at low temperatures, the possibility of using green solvents, and a short extraction time. However, it also presents limitations, as it remains underexplored in microalgal research, particularly regarding the use of enzymes mixture in EAE [113], while in terrestrial plant research the use of enzymes mixtures for EAE is well documented [113].

Additionally, EAE requires precise control over parameters such as pH and temperature, which can affect the extraction efficiency of bioactive compounds. Therefore, optimization of these parameters is crucial [104,108].

EAE is widely used for lipid extraction from marine, brackish, and fresh water microalgae [103,106,110,113]. It is also applied in protein hydrolysis to produce peptides [114], and in the recovery of carotenoids [111].

### 2.7. Green Solvents Extraction

In addition to green extraction techniques, green solvents can be used for the extraction of bioactive compounds. These include agro- or bio-solvents, which are potential alternatives to conventional organic solvents [42]. Green solvents are biodegradable, non-toxic, and non-flammable, sometimes derived from natural sources [42]. They can be used either alone or in combination with extraction techniques, although research in the field of solvent screening remains limited [115]. Extraction using a green solvent can be performed at room temperature by soaking the matrix for the desired extraction time [116,117,118,119]. In Prat et al. [120], all the solvents presented in this section are well classified and recommended as green solvents.

Ethanol is considered a bio-solvent when derived from the fermentation of natural sugar-rich materials, despite its flammability [42]. However, ethanol can also be synthesized petrochemically, in which case it should no longer be qualified as a bio-solvent. Ethanol is inexpensive and has a high affinity for lipid extraction, often achieving yields comparable to or higher than those obtained using organic solvents [118]. Ethyl acetate (EtOAc) is another green solvent with minimal impact on human health [115]. 2-Methyltetrahydrofuran (2-MeTHF), produced from renewable resources, exhibits extraction efficiency nearly identical to that of chloroform [115]. Terpenes, such as limonene, offer a promising alternative to organic solvents due to their natural origin (citrus fruits), low cost, and low toxicity [121]. Additionally, its extraction yields are very similar to those obtained with hexane, as its dielectric constant is close to that of hexane [83]. Water, at ambient temperature and pressure, is a highly polar solvent. However, by modifying its temperature and pressure, its dielectric constant can be reduced to facilitate the extraction of water-soluble compounds such as proteins, sugars, and organic acids [42]. Its use is well-documented in subcritical water extraction (SWE), as discussed in Section 2.5, and it is considered the safest, least expensive, and non-toxic solvent [79]. Ionic liquids (ILs) are also considered an alternative to organic solvents due to their non-volatile and non-flammable nature, classifying them as green solvents [122]. However, in some cases, ILs exhibit a greater life cycle environmental impact compared to conventional organic solvents [37,123]. Another emerging alternative to both ILs and organic solvents is switchable solvents (SSs). These are non-volatile liquids that can transition from a hydrophobic to a hydrophilic state in response to changes in temperature, pH, or the addition/removal of a gas [37,124], making SSs particularly promising for the extraction of both polar and non-polar compounds. Deep eutectic solvents (DESs) have emerged as alternatives to conventional organic solvents, exhibiting simple preparation, low toxicity and high biodegradability, but studies have mostly focused on the extraction of lipids and carotenoids from microalgae [125]. DESs are also promising green alternatives for the pretreatments of microalgal biomass, which is a crucial step in the extraction of bioactive compounds by rupturing the cell wall [125]. DESs consist of a mixture of two or more solid components (hydrogen band acceptors such as choline chloride and hydrogen band donors such as urea) allowing a melting point at least 100 °C lower than the starting materials. DESs are found to be cheaper than ILs, which emphasize their use in the industry and the interest by the scientific community [126]. Natural deep eutectic solvents (NaDESs) are also highly regarded due to their profusely availability in nature, and renewable properties, consisting of cholinium chloride, natural carboxylic acids, sugars, and amino acids [126,127]. The main limitation of DESs and NaDESs is the choice of the pretreatment that needs to maximize the recovery of the bioactive compounds [127]. A fluorous solvent (FS) such as perfluorocyclohexane is a green solvent mostly used in the extraction of lipids from microalgal biomass. FSs exhibit several advantages: FSs are inert in nature, non-toxic, and facilitate phase separation as they consist of an aqueous phase and a non-aqueous phase (rich in fluor). However, their main limitations are the dependance to temperature, with which their density and viscosity can change [59]. Thermomorphic systems (TMSs) consist of different components (e.g., water, buffers, ILs, DESs) that can be altered via the temperature, therefore changing the miscibility of the solution and allowing the recovery of polar bioactive compounds in the aqueous phase and non-polar compounds in the non-aqueous phase [128]. However, to our knowledge, TMSs have not yet been tested to extract compounds from microalgal biomass.

These solvents enable the extraction of lipids and carotenoids from marine and freshwater microalgae species [59,83,115,117,118,121,125]. However, further research is needed to better characterize the extracted compounds and expand the range of target compounds.

## 3. Green Extraction Techniques Compared with Conventional Techniques

Conventional extraction methods rely on organic solvents such as methanol, chloroform, and hexane, which pose several challenges. At the pilot or industrial scale, these solvents require high energy consumption, are flammable, environmentally unfriendly, and can be hazardous to human health [37]. In Prat et al. [120], hexane and chloroform are classified as highly hazardous, with concerning environmental and health scores. Nevertheless, they are still widely used for extracting bioactive compounds from microalgae across various application sectors.

Among conventional extraction techniques, the Folch method is the most used for lipid extraction from microalgae, followed closely by the Bligh and Dyer method [37]. Both techniques involve simple procedures but rely on hazardous solvent mixtures (chloroform/methanol and chloroform/methanol/water, respectively). Soxhlet extraction, on the other hand, requires a long extraction time, leading to high energy consumption, and is often used with organic solvents for the extraction of lipids and carotenoids [37,129].

In Table 2, green extraction methods, compared to conventional techniques, show promising potential, often achieving higher yields. The mass of microalgae used varies, whether dry or wet (column biomass in Table 2), further complicating an effective comparison. The yields were calculated based on the biomass used, then reported in mg/g of dry biomass, in g/100 g of dry biomass, or % of dry biomass, complicating an efficient comparison due to a lack of standardization in the report of results.

**Figure 1:**
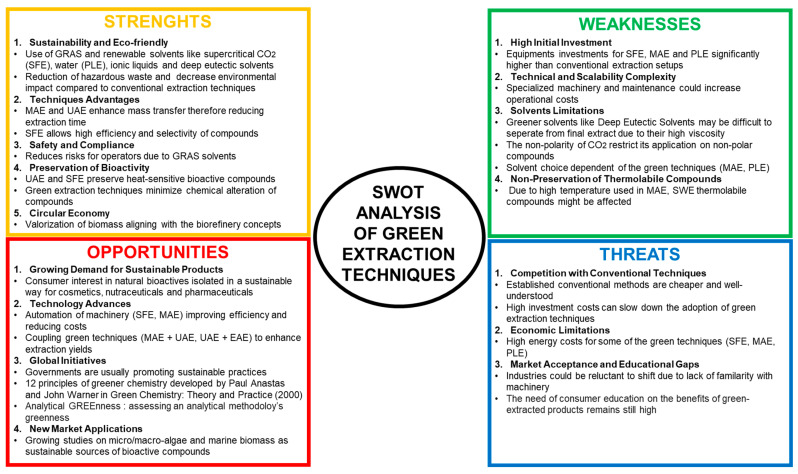
SWOT (strengths, weaknesses, opportunities, and threats) of green extraction techniques.

Green extraction techniques are emerging as sustainable methods in the recovery of bioactive compounds, prioritizing sustainability (use of renewable biomass, alternative solvents, production of co-product), safety (final extract without contaminants), and efficiency, aligning with the six principles of green extraction of natural products. Figure 1 aims to highlight the positioning of green extraction techniques. The SWOT (strengths, weaknesses, opportunities, and threats) assessment highlights the potential of green extraction techniques as eco-friendly and sustainable alternatives to conventional methods. Their strengths in sustainability, bioactivity improvement, and global initiatives are offset by economic and scalability challenges. However, technological innovations and rising demand for sustainable products by consumers are driving their adoption. Addressing the weaknesses in scalability and market applications could accelerate their integration at industrial scale by reducing operation costs through the optimization of the green extraction processes and training more professionals.

## 4. Compounds Extracted from Microalgae with Green Extraction Techniques

Studies on microalgae extraction by using green extraction techniques have been reported for both freshwater and marine species, with a total of 19 species of microalgae studied, of which 12 marine and 7 freshwaters. Main data are available on the marine species *Chlorella* sp. and *Nannochloropsis* sp., and the freshwater species *Arthrospira platensis*.

Primary bioactive compounds extracted were lipids and pigments, respectively extracted for biodiesel production and the sectors of food and pharmaceutics. The three most studied species, i.e., *Chlorella* sp., *Nannochloropsis* sp., and *Arthrospira platensis*, have all been subjected to each of the green extraction techniques discussed in this review. Depending on the extraction technique and the microalgal species, the compounds extracted may vary. Lipids are predominantly extracted across all extraction methods for *Nannochloropsis* sp., whereas for *Chlorella* sp., both pigments and lipids are extracted in roughly equal proportions, depending on the technique used, highlighting the selective nature of certain green extraction methods regarding the compounds targeted. For *Arthrospira platensis*, pigments are the most commonly extracted compounds across all techniques.

Even if the yield units reported are sometimes different (e.g., % or grams), it seems that higher yields of lipids and pigments have been obtained by SFE-CO_2_ and PLE extraction. Moreover, yield from PLE can be obtained by combining the technique with green solvents, making it a highly promising technique. SWE is primarily used for the extraction of proteins and carbohydrates, for which it provides better yields compared to other green extraction techniques. Detailed information is reported in Table 3.

## 5. Discussion

The extraction methods reviewed in this paper present specific advantages and limitations. Conventional extraction techniques remain widely used within the scientific community due to their simplicity, relatively high yields, and established practices. However, growing environmental concerns are driving a shift towards alternative methods that minimize or eliminate the use of organic solvents. Among these, SFE-CO_2_ is a promising green alternative that enables selective extractions but requires a high initial investment. MAE, UAE, and PLE significantly reduce solvent consumption, but their efficiency is dependent on solvent choice, which varies depending on the target bioactive compounds. SWE has gained considerable attention due to its environmentally friendly approach, using water as a solvent, but it is not recommended for thermolabile molecules. Conversely, EAE is a suitable alternative for thermolabile compounds but relies on the effective disruption of the microalgal cell wall.

In the process of discovering marine natural product drugs, once the bioactive compounds are identified and their structure characterized, the material can either be used as-is or modified. In either case, the compound must be obtained in larger quantities, requiring a scale-up process to potentially supply enough material for clinical trials [148]. Few, if any, studies have been conducted on the scale-up of green extraction methods for microalgae due to non-availability of proper optimized production systems, proper microalgal strains, and optimal growth parameters for enhanced biomass yields [25,149], though this has been widely documented for terrestrial plants [150,151,152,153,154,155]. Large-scale investigations for terrestrial plants have shown that UAE is one of the best non-thermal green extraction techniques, and MAE shows promising results, though few studies have been conducted [152]. SFE-CO_2_ is commonly used in laboratory-scale extraction and optimization of compounds from terrestrial plants, and it demonstrates higher to similar yields in oil extraction at pilot-scales [152]; for example, the extraction of oil from seed rape reached 100.3 g per kWh at pilot-scale (extractor of 16 L against less than 1 L at laboratory scale) close to the oil recovery reached at laboratory scale, 97.4 g per kWh. While these findings could be applicable to microalgae, a major limitation is the amount of biomass required for these extractions. Working with unicellular organisms requires greater biomass production than working with terrestrial plants. Moreover, the utilization of SFE, particularly in the production of biodiesel from microalgal biomass, requires high investment costs which can cool down industries from investing and large amounts of biomass to potentially be able to be marketed. According to Tabernero et al., 2012 [156], the production of 10,000 ton/year of biodiesel from heterotrophic microalgae would not be viable due to the excessive number of photobioreactors required and the loss of oil in the extraction process, emphasizing the need for the optimization of green extraction techniques and microalgal biomass production to reach fully commercialization. Another limitation in the production of bioactive compounds from microalgae is the cost of the total production process. According to Guedes et al. [149], natural astaxanthin from *Haematococcus* is more expensive to produce than synthetic astaxanthin (USD 1000/kg). To be as competitive as synthetic astaxanthin, natural astaxanthin should be produced at less than USD 30/kg, which is not the case even with the current technologies.

One of the major limitations in comparing the yields of bioactive compounds from microalgae across different extraction techniques is the non-standardized use of units for reporting results. In this review, yields are expressed in various ways, such as percentage of dry weight, grams of extract per 100 g of dry biomass, and percentage of recovery, among others, making effective comparison challenging. Additionally, the amount of microalgal biomass used per extraction technique is another factor influencing yield outcomes. This parameter varies significantly between studies, ranging from a few milligrams to several grams, and is not standardized for each microalgal species. Not only does biomass quantity and moisture content affect yield, but it is also influenced by the microalgal species, cultivation conditions, and pretreatment methods often applied to enhance extraction efficiency, and thus biological activity. For instance, diatoms are frequently subjected to pretreatments due to their frustule, a silica shell encasing the cell, to optimize the extraction of bioactive compounds [157,158]. However, the use of pretreatments (e.g., ultrasound, freeze-drying, enzymatic digestion) may introduce additional economic and environmental costs, such as increased energy consumption and the use of organic solvents.

Green extractions of bioactive compounds from microalgae are influenced by upstream conditioning and biomass stabilization. Indeed, the optimization of upstream conditions (growth optimization, harvesting, and pretreatment) and biomass stabilization will influence the yield and efficiency of the bioactive compounds [159,160].

The type of bioactive compounds and their yield is influenced by the growth conditions [161]. Microalgae can be classified as autotrophs, mixotrophs and heterotrophs according to the source of carbon they require therefore influencing the bioactive compounds produced. Indeed, in heterotrophic condition, literature showed a higher lipid production (four times higher) compared to autotroph conditions [162]. Other growth factors, such as light, nutrient, salinity, and temperature, influence the growth of the microalgae, and therefore the production of bioactive compounds [163].

The harvesting part of the upstream conditions counts for 20–30% of the microalgal biomass cost, emphasizing the need to understand which harvesting technique is more suitable for high-valued metabolites [164]. Biomass harvesting is also dependent of the microalgal species; for example, diatoms are usually harvested at the end of their stationary phase where it has been shown to yield more secondary metabolites [161,165]. For biodiesel production from microalgae, coagulation and flocculation seem to be the more suitable methods [159,164], while for high-valued products from microalgae (e.g., pigments), centrifugation followed by coagulation and flocculation are the more suitable harvesting techniques [164]. The pretreatment of the algal biomass (e.g., ultrasound, bead milling, hydrolysis) is crucial in the disturbance of the cell wall for an effective release of the compounds and is dependent on the robustness of the specific cell wall of microalgae species [127,166,167]. The pretreatments often use before the extraction with NaDESs are MAE, UAE, and EAE due to their economical, efficient, and fast advantages, achieving up to 90% of recovery [127]. However, according to the extraction use, some techniques do not necessarily need pretreatment, such as UAE or MAE (Table 3), because the microwave and ultrasound energy are enough to disturb the cell wall and achieve an efficient recovery of compounds.

To prevent deterioration and the quality of the bioactive compounds, stabilization is a crucial step. However, the stabilization methods employed can affect the extraction yields, by being compound-specific, and properties of the compounds highlighting the importance to understand the effect of stabilization method on the extraction techniques [160]. The stabilization of microalgae biomass can be classified in three methods: physical, chemical, and biological. Physical stabilization are diverse such as drying methods lowering the enzymatic activities of the biomass and preventing microbial growth [160]. Ultrasound is also known to be a non-thermal physical stabilization method by damaging the microbial cell walls therefore preventing microbial growth [160]. Chemical stabilization methods also prevent microbial growth and limit enzymatic activities by adding acid/base solutions to the microalgae biomass to lower or increase its pH [160]. Biological stabilization such as fermentation or the addition of enzymes are also techniques used in the stabilization of the biomass [160].

Despite the difficulty in comparing the extracted compounds, particularly in Table 3, pigments and lipids are the most extracted compounds. Pigments are primarily extracted for applications in the cosmetic, nutrition, and pharmaceutical sectors, while lipids are mainly used for biofuel production. *Chlorella* sp., *Nannochloropsis* sp., and *Arthrospira platensis* are the most studied microalgae in this review. Indeed *Chlorella* sp., *Nannochloropsis* sp., and *Arthrospira* sp. are the most studied species in literature for the production of bioactive compounds [25]. While over 200,000 microalgae species have been discovered, only a few are used for industrial applications [168]. The main reason is safety regulations, as compounds extracted from untested species have to overcome socio-ethnological and toxicity-related issues before possible market applications [25]. *Chlorella* has been extensively studied for the extraction of both lipids and pigments. This is largely due to its exceptionally high chlorophyll content, one of the highest found in nature [169,170], as well as its classification among unicellular green microalgae with a high lipid content [171,172], supporting its frequent evaluation for lipid and pigment extraction in this review. *Chlorella* is valued for its versatility in cultivation, as it can grow under both autotrophic and heterotrophic conditions, similar to *Arthrospira platensis*, making it an ideal candidate for large-scale production with reduced contamination risks and higher productivity [173]. UAE is one of the most commonly used methods for *Chlorella*, primarily due to its multi-layered cell wall, which requires efficient disruption to release intracellular compounds [166,174] while preserving potentially thermolabile bioactive compounds. *Nannochloropsis* has been extensively studied for its high lipid content, which ranges from 30% to 60% of its dry weight, as well as for its fatty acid composition, which is particularly relevant for biodiesel production [175]. It is important to highlight that the use of microalgae in biodiesel production must be optimized to ensure both economic and environmental sustainability, with particular emphasis on the green extraction techniques employed for lipid recovery from *Nannochloropsis* sp. In this review, various green extraction methods have been applied, showing promising results in terms of lipid and fatty acid yields. However, comparison between green and conventional techniques and yield among green techniques remains challenging due to inconsistencies in reporting units and the biomass loading used in different studies. Furthermore, pretreatment is commonly required for lipid extraction from *Nannochloropsis* due to its robust cell wall. The coupling of green extraction techniques with environmentally friendly cell disruption methods, such as ultrasound, has demonstrated increased lipid yields, supporting the development of more sustainable processes for biodiesel production from *Nannochloropsis* sp. [167]. Finally, *Arthrospira platensis* is primarily studied for its applications in the food and pharmaceutical sectors due to its production of secondary metabolites such as phycocyanin, a blue pigment, which has demonstrated nutritional value when included in the diet, along with a wide range of medical effects including antioxidant, antidiabetic, neuroprotective, and antimicrobial properties [176]. This review highlights the extraction of primarily pigments from *Arthrospira platensis*. As pigments are known to be thermolabile, green extraction techniques that avoid high temperatures are preferred, such UAE, PLE, or EAE, which have shown to be efficient for pigment recovery in Table 3 [177]. Since *Arthrospira platensis* is a microalga that produces several valuable metabolites, it is also worth exploring techniques that allow for the efficient extraction of lipids, pigments, and vitamins at the same time, such as SFE-CO_2_ and MAE [138].

Initiatives in the use of greenness assessment criteria to determine whether an analytical method is more or less sustainable have been developed, such as the Analytical GREEnness calculator [178]. This tool takes the 12 criteria of green analytical chemistry and transforms them in order to give a score between 0–1 to minimize environmental impact in laboratory processes. Analytical GREEnness is a metric system that is comprehensive, flexible (possibility to assign weights), and easy to interpret and perform.

## 6. Conclusions

In this review, green extraction techniques generally yield higher results compared to conventional methods. In rare cases, where conventional extraction achieves slightly better yields, the difference does not exceed 3% differences (Table 2). This highlights the potential of green extraction methods for recovering bioactive compounds for applications across various sectors. Among the studies analyzed, the most extracted compounds using green extraction techniques are lipids, which are predominantly obtained through SFE-CO_2_ for biofuel production, and many works focus on comparing green and conventional methods for lipid extraction from microalgae. While green extraction methods for pigments, carbohydrates, and proteins are under development and are relatively well-documented, they remain less extensively studied compared to lipid extraction for biofuel production from microalgae. Further investigation is needed to compare the extraction of other bioactive compounds such as vitamins, carbohydrates, and proteins. Depending on the green extraction technique employed, both the yield and the composition of bioactive compounds of the extracts vary. In this review, lipids and pigments are the most frequently extracted bioactive compounds across all the techniques discussed. The highest yields of both lipids and pigments are obtained using SFE-CO_2_, MAE, and PLE. These yields, however, are highly dependent on the quantity and characteristics of the microalgal biomass used. The choice of extraction techniques will always be dependent on the objectives and conditions: biomass cell specificity, biomass availability, choice of solvent, targeted compounds, type of industry application, and economic valorization goal. For future research perspectives, the optimization of green extraction methods is necessary, particularly in the use of green solvents in already green techniques to maximize the yields of bioactive compounds. Green extraction methods show promising results at the laboratory scale; however, scaling up to an industrial level remains limited due to high investment and production costs. Compared to conventional extraction methods, green extractions exhibit a lower environmental impact. However, further research on the overall production process, including analyses of carbon footprint, water and energy consumption, and waste management, is an important research perspective to ensure the sustainability of these emerging green technologies. This review highlights the need to further explore green extraction techniques of bioactive compounds from microalgae trying to reduce the production costs since they promise more advantageous results in terms of yield but also in terms of sustainability than conventional methods.

## Figures and Tables

**Table 1:** Enzymes used in EAE on microalgae.

Microalgae	Enzyme	Optimum Temperature (°C)	Optimum pH	Compounds Recovery	References
*Chlorella* *vulgaris* *Scenedesmus* *dimorphus* *Nannochloropsis* sp.	Cellulase	55	4.8	Lipids (15.11% of dry biomass) Lipids (10.62% of dry biomass) Lipids (15.98% of dry biomass)	[110]
	Neutral Protease	50	7.0		
	Alkaline Protease	55	8.5		
	Trypsin	37	8.0		
	Snailase	37	5.8		
*Arthrospira platensis*	Lysozyme	37	6.8	Allophycocyanin (32.27 ± 1.90% of dry biomass)	[111]
*Nannochloropsis salina*	Rhizomucor miehei lipase	40	-	Lipids (0.416 ± 0.008 g/g of dry biomass)	[112]
*Chlorella* *vulgaris*	Cellulase	55	4.8	Lipids (18% of dry biomass)	[103]
	Lysozyme	55	-	Lipids (24% of dry weight)	
	Snailase	37	5.8	Lipids (22% of dry biomass)	
*Scenedesmus* sp.	Mixture of cellulase, hemicellulase, and pectinase	30/50 °C	3.5/4.5	Oil recovery (86.1% g/g of dry biomass)	[113]

**Table 2:** Comparison of yields obtained by conventional extraction techniques and green extraction techniques.

Microalgae	Extraction Method	Solvent	Biomass	Yield	References
*Chlorella* sp.	Switchable Solvent	EBA ^b^ (1:1 water)	Wet	Oil (12.35 ± 3.18% dw ^a^)	[130]
	Switchable Solvent	DMCHA ^c^	Wet	Oil (13.30 ± 0.42% dw)	
	Organic solvent	Hexane	Dry	Oil (9.38 ± 0.73% dw)	
*Chlorella* sp.	UAE	Media	Wet	Carbohydrates (36.85 ± 1.35 g/100 g dw)	[86]
	Conventional	Distilled Water	Dry	Carbohydrates (9.06 ± 0.05 g/100 g dw)	
*Chlorella vulgaris*	MAE	Water/[Omim][OAc] 2.5%	Dry	Lipids (19.2% dw)	[60]
	Bligh and Dyer	Methanol/chloroform (2:1)	Dry	Lipids (10.9% dw)	
*Chlorella vulgaris*	PLE	Ethanol 90%	Dry	Lutein (3.78 ± 0.19 mg/g dw) ß-carotene (0.50 ± 0.25 mg/g dw) Chlorophyll a (9.63 ± 0.65 mg/g dw) Pheophorbide a (0.01 ± 0.00 mg/g dw)	[71]
	Soxhlet	Ethanol 90%	Dry	Lutein (3.42 ± 0.11 mg/g dw) ß-carotene (0.26 ± 0.09 mg/g dw) Chlorophyll a (3.32 ± 0.30 mg/g dw) Pheophorbide a (5.15 ± 0.59 mg/g dw)	
	Maceration	Ethanol 90%	Dry	Lutein (2.97 ± 0.31 mg/g dw) ß-carotene (0.08 ± 0.01 mg/g dw) Chlorophyll a (4.26 ± 0.53 mg/g dw) Pheophorbide a (0.85 ± 0.09 mg/g dw)	
*Chlorella vulgaris*	SWE	Water/ethyl acetate	Wet	Lipids (53.40% dw)	[131]
	Bligh and Dyer	Chloroform/methanol	Dry	Lipids (10.4% dw)	
*Galdieria phlegrea*	SFE-CO_2_	CO_2_	Wet	Lipids (184 ± 5 mg/g dw)	[132]
	Organic solvent	Chloroform/methanol/sodium chloride	Dry	Lipids (164 ± 6 mg/g dw)	
	PLE	Ethanol	Wet	Carotenoids (89 ± 6 mg/g dw)	
	Organic solvent	Acetone	Dry	Carotenoids (100 ± 5 mg/g dw)	
*Nannochloropsis gaditana*	Switchable Solvent	DMCHA2	Wet	Lipids (57.9 ± 1.3% dw)	[130]
	Bligh and Dyer	Chloroform/methanol/water	Dry	Lipids (45.1 ± 0.9% dw)	
*Nannochloropsis gaditana*	MAE	Methanol (1:10)	Dry	Fatty acids (14.36 ± 0.41% dw)	[61]
	UAE	Methanol (1:10)	Dry	Fatty acids (14.11 ± 0.40% dw)	
	Conventional	Methanol (1:10)	Dry	Fatty acids (13.59 ± 0.39% dw)	
*Nannochloropsis oculata*	UAE	Medium	30% Dry	FAMEs ^d^ (0.2 ± 0.03% dw)	[85]
	Bligh and Dyer	Methanol–chloroform (2:1)	20% Dry	FAMEs (5.7% dw)	
*Nannochloropsis* sp.	SFE-CO_2_	CO_2_	Dry	Lipids (34 g/100 g dw) Pigments (38 mg/100 g dw)	[46]
		CO_2_ + 20% ethanol		Lipids (45 g/100 g dw) Pigments (100 mg/100 g dw)	
	Soxhlet	Hexane	Dry	Lipids (40.7% dw)	
	Bligh and Dyer	Methanol/chloroform/water (10:5:4)	Dry	Lipids (25.3% dw)	
*Nannochloropsis* sp.	MAE	NaCl solution	Dry	Lipids (16.1% dw)	[133]
	Bligh and Dyer	Chloroform/methanol/water (1:2:0.8)	Dry	Lipids (18% dw)	
	Soxhlet	Hexane	Dry	Lipids (4.5% dw)	
*Neochloris oleoabundans*	Bligh and Dyer with Switchable Solvent	EBA	Wet	Lipids (47% dw)	[134]
	Bligh and Dyer	Methanol/chloroform (2:1)	Dry	Lipids (13% dw)	
*Phaeodactylum tricornutum*	SFE-CO_2_	CO_2_	Dry	Lipids (25% dw)	[135]
	Folch	Chloroform/methanol/water	Dry	Lipids (28% dw)	
*Spirulina platensis*	MAE	Methanol/hexane (1:2)	Dry	Lipids (12.53% dw)	[136]
	Soxhlet	Hexane	Dry	Lipids (1.293% dw)	
*Spirulina platensis*	UAE	Ethanol	Dry	Phycocyanin (13.61% dw)	[137]
	Conventional	Ethanol	Dry	Phycocyanin (11.13% dw)	

^a^ dw: dry weight, referring to dry algae biomass; ^b^ EBA: *N*-ethylbutylamine; ^c^ DMCHA: N,N-dimethyl-cyclohexylamine; ^d^ FAMEs: Fatty Acids Methyl Esters.

**Table 3:** Compounds extracted from microalgae with green extraction techniques.

Microalgae	Techniques	Methodology and Solvents	Environment	Compounds and Yield	Application Field	Ref.
*Anabaena planctonica*	PLE	T: 200 °C P: 20.7 MPa Time: 15 min Solvent: Limonene:ethanol (1:1)	Freshwater	Total Yield (3.1 ± 0.5% *w*/*w*) ^a^ Lipids (6.0 ± 1.1% *w*/*w*) ^b^ Gamma-linolenic acid (1.3 ± 0.4% *w*/*w*)	Biodiesel production	[83]
*Arthrospira platensis*	SFE-CO_2_	T: 60 °C P: 450 bar Time: 50 min Co-solvent: Ethanol/water 96% (*v*/*v*)	Freshwater	Carotenoids (283 ± 0.10 μg/g dw ^b^) Tocopherols (5.01 ± 0.05 μg/g dw) Fatty acids (34.76 ± 0.08 mg/g dw)		[138]
	MAE	T: 50 °C P: 1 bar Time: 15 min Power: 400 W Solvent: Methanol/ethyl acetate/light petroleum (1:1:1 *v*/*v*)		Carotenoids (629 ± 0.13 μg/g dw) Tocopherols (2.46 ± 0.09 μg/g dw)		
		T: 70 °C P: 1 bar Time: 15 min Power: 400 W Solvent: Methanol/ethyl acetate/light petroleum (1:1:1 *v*/*v*)		Fatty acids (15.88 ± 0.06 mg/g dw)		
		T: - P: - Time: 40 min Power: 600 W Solvent: Methanol-hexane solvent (1:2)		Lipids yield (% dw) 12.53%	Biodiesel Production	[136]
	PLE	T: 40 °C P: 103.4 bar Time: 15 min Solvent: DMSO (100%) Magnetic stirring		ß-carotene (29.11 mg/L) Myxoxanthophyll (1.54 mg/L) Zeaxanthin (24.93 mg/L)	Food Industry	[77]
		Pretreatment T: 25 °C P: 1500 psi Time: 15 min Solvent: Water		Proteins Yield (8% dw)	Food and Research sectors	[82]
		T: 200 °C P: 20.7 MPa Time: 15 min Solvent: Limonene:ethanol (1:1, *v*/*v*)		Total Yield (17.6 ± 0.2% *w*/*w*) Lipids (33.7 ± 2.4% *w*/*w*) Gamma-Linolenic acid (7.3 ± 0.5% *w*/*w*)	Biodiesel Production	[83]
	UAE	T: 52.5 °C Time: 42 min Power: - Solvent: Ethanol		Phycocyanin (14.94 ± 0.21% g/g) Anti-oxidant activity EC50 (86.3 ± 1.65 mg/mL)	Food, Pharmaceutical, Cosmetic Industries	[137]
	SWE	T: 180 °C P: 20 bar Flow rate: 6 mL/min Time: 14 s		Protein Yield (60.2 ± 0.7% dw) Total Phenolic Compounds (≈25 mg GAE/L) TEAC ^c^ (≈5 µmol TEAC/L)	Microalgae biorefinery	[139]
		T: 210 °C P: 20 bar Flow rate: 5 mL/min Time: 17 s		Carbohydrate Yield (>100% dw) Total Phenolic Compounds (40 mg GAE/L) TEAC (≈7 µmol TEAC/L)		
	EAE	Ultrasonication Enzyme: Lysozyme T: 27 ± 2 °C pH: 6.8 Time: 4 h Intermittent stirring		Allophycocyanin (44.08 mg/g dw)		[111]
*Chlorella pyrenoidosa*	SWE	T: 270 °C Time: 10 min	Marine	Proteins Yield (18.773% dw) Carbohydrates Yield (1.34% dw)	Biproduct from microalgae	[101]
*Chlorella vulgaris*	SFE-CO_2_	T: 60 °C P: 30 MPa Time: 120 min Co-solvent: Ethanol 7.5% (*v*/*v*)	Marine	Chlorophyll a (37.50%) Chlorophyll b (2.83%) Lutein (19%) β-Carotene (2.93%)	Pigments for foods and use in pharmaceutical sector	[140]
	MAE	T: 60 °C P: - Time: 5 min Power: 700 W Solvent: 2.5% of hydrophilic Ionic Liquids [Omim][OAc]		Lipids yield (20% dw) FAMEs ^d^ (8.3% total lipid weight)		[60]
	PLE	T: 200 °C P: 1500 psi Time: 20 min Solvent: Water		Total yield (39.31% dw) TEAC (1.913 ± 0.099 mmol Trolox/g extract)	Antioxidant and antimicrobial activities for food industries	[81]
		T: 200 °C P: 1500 psi Time: 20 min Solvent: Ethanol		Total yield (36.43% dw) TEAC (0.196 ± 0.003 mmol Trolox/g extract)		
		T: 50 °C P: 1500 psi Time: 20 min Solvent: Acetone		Total yield (0.94% dw) TEAC (0.479 ± 0.009 mmol Trolox/g extract)		
		T: 40 °C P: 103.4 bar Time: 15 min Solvent: DMSO (100%) Magnetic stirring		Lutein (0.82 mg/L) ß-carotene (0.2 mg/L) α-carotene (0.41 mg/L)	Food Industry	[77]
		T: 160 °C P: 1500 psi Time: 30 min Solvent: Ethanol (90%)		Lutein (3.78 ± 0.19 mg/g of extract) ß-carotene (0.50 ± 0.25 mg/g of extract) Chlorophyll a (9.63 ± 0.65 mg/g of extract) Pheophorbide a (0.01 ± 0.00 mg/g of extract)	Food and Pharmaceuticals industries	[71]
	UAE	T: 25 °C Time: 25 min Solvent: Ethanol Magnetic stirring		Total yield (4.79% dw) TEAC (0.588 ± 0.021 mmol Trolox/g extract)	Antioxidant and antimicrobial activities for food industries	[81]
		Enzymatic pretreatment T: 37.7 °C Time: 162 min Solvent: Ethanol (35.6 mL/g)		Lutein (3.36 ± 0.10 mg/g of wet biomass)	Food and Pharmaceuticals industries	[92]
		T: - Time: 30 s on, 5 s off (20 min) Power: 600 W Solvent: -		Lipid concentration (≈15% dw)	Biodiesel Production	[103]
	SWE	T: 200 °C P: 1500 psi Time: 20 min		Total yield (>55% dw) Total phenols (32.91 ± 4.47 mg gallic acid/g extract)	Recovering antioxidant compounds from natural ressources	[97]
		T: 160 °C P: 80 bar Time: 30 min Co-solvent: Ethyl Acetate		Lipids (53.40% dw)	Biorefinery	[131]
	EAE	Enzyme: Cellulase Concentration: 5.00 mg/L T: 55 °C Time: 10 h		Lipid concentration (≈25% dw)	Biodiesel Production	[103]
		Enzyme: Lysozyme Concentration: 5.00 mg/L T: 55 °C Time: 10 h		Lipid concentration (≈22% dw)		
		Enzyme: Snailase Concentration: 5.00 mg/L T: 37 °C Time: 2 h		Lipid concentration (≈7% dw)		
		Pretreatment Enzyme: Snailase + trypsin T: 37 °C pH: 4 Time: 12 h		Lipid recovery (49.82% lipid content)	Biodiesel Production	[110]
*Cylindrotheca closterium*	MAE	T: 56 °C P: 101.325 Pa Power: 50 W Time: 5 min	Marine	Fucoxanthin (4.24 ± 0.09 μg/mg per extract) Chlorophyll a (8.65 ± 0.29 μg/mg per extract)	Food, Health and Biotechnology applications	[65]
	UAE	T: 8.5 °C P: 101.325 Pa Time: 10 min Power: 12.2 W Solvent: Acetone		Fucoxanthin (4.49 ± 0.08 μg/mg per extract) Chlorophyll a (4.95 ± 0.27 μg/mg per extract)		
*Dunaliella tertiolecta*	MAE	T: 56 °C P: 101.325 Pa Power: 50 W Time: 5 min	Marine	Chlorophyll a (>4 μg/mg per extract) Chlorophyll b (>1 μg/mg per extract) ß-carotene (>1 μg/mg per extract)		
	UAE	T: 8.5 °C P: 101.325 Pa Time: 10 min Power: 12.2 W Solvent: Acetone		Chlorophyll a (>4 μg/mg per extract) Chlorophyll b (>1 μg/mg per extract) ß-carotene (>1 μg/mg per extract)		
*Galdieria phlegrea*	SFE-CO_2_	Pretreatment T: 60 °C P: 350 bar Time: 100 min	Freshwater	Lipids (184 ± 5 mg/g dw)	Nutraceutical, pharmaceutical, foods, and biofuel industries	[132]
	PLE	Pretreatment T: 50 °C P: 100 bar Time: 30 min Solvent: Ethanol		Carotenoids (89 ± 6 mg/g dw)		
*Haematococcus pluvialis*	SFE-CO_2_	T: 50 °C P: 4500 psi Time: 20 mi8 Co-solvent: Ethanol 99.5%	Freshwater	Astaxanthin (10.92 mg/g dw)	Antioxidant for foods and cosmetics	[54]
		T: 343 K P: 40 MPa Time: 30 min 8 Co-solvent: Ethanol 7.5% (*v*/*v*)		Astaxanthin (80.6% per extract)	Pigments for foods, Antioxidant for foods and cosmetics	[141]
	PLE	Pretreatment T: 40 °C P: 10.34 MPa Time: 20 min Solvent: Ethanol		Total Yield (3.7 g/100 g dw) TEAC (0.426 ± 0.004 mmol Trolox/g extract)	Food and pharmaceuticals industries	[78]
		Pretreatment T: 200 °C P: 10.34 MPa Time: 20 min Solvent: Ethanol		Total Yield (37.1 g/100 g dw) TEAC (0.287 ± 0.005 mmol Trolox/g extract)		
	UAE	T: 45 °C Time: 60 min Power: 18.4 W Solvent: Acetone		Astaxanthin (60% dw)	Pigments for foods, Antioxidant for foods and cosmetics	[142]
	SWE	Pretreatment T: 200 °C P: 1500 psi Time: 20 min		Total Yield (33% dw) MBC ^e^ (5.0 mg/mL) MFC ^f^ (5.5; 15 mg/mL) TEAC (1.974 ± 0.053 mmol TE/g d.m.)	Recovering antioxidant and antimicrobial compounds	[98]
*Nannochloropsis gaditana*	SFE-CO_2_	T: 50 °C P: 350 bar Time: 135 min	Marine	Proteins (42.73% dw) Carbohydrates (27.67% dw) Lipids (11.83% dw) SFAs ^g^ (32.20% lipids dw) MUFAs ^h^ (27.21% lipids dw) EPA ^i^ (33% lipids dw) Other PUFAs ^j^ (7.59% lipids dw)	Aquaculture: feed supplements	[44]
	MAE	T: 60 °C P: - Time: 10 min Power: 1000 W Solvent: Methanol (1:10 ratio)		Lipid yield (29.7% dw)	Biofuel production	[61]
		T: 90 °C P: - Time: 10 min Power: 1000 W Solvent: Methanol (1:10 ratio)		Lipid yield (40% dw)		
	UAE	T: 50 °C–60 °C Time: 20 min Power: 100 W Solvent: Methanol (1:10 ratio)		Lipid yield (36.2% dw)	Biofuel production	
	SWE	T: 236.54 °C P: 1500 psi Time: 13.95 min		Lipid Yield (13.405% dw) EPA (15.040% of total FAMEs)	Pharmaceuticals sectors	[99]
*Nannochloropsis oculata*	PLE	T: 60 °C P: 10–12 MPa Time: 5 × 10 min Solvent: Ethanol (vol.96%)	Marine	Total Yield (36 ± 4% dw) Fatty acids (16.7 ± 0.6% dw) EPA (3.7 ± 0.1% dw)	Biorefining	[84]
	UAE	T: - Time: 25 min Power: 1500 W		FAMEs (2606.0 μg/g dw)	Biodiesel production	[85]
*Nannochloropsis salina*	SWE	T: 220 °C Time: 20 min	Marine	Lipid Yield (27 ± 1.3% dw)	Biodiesel production	[100]
*Nannochloropsis* sp.	SFE-CO_2_	Mechanical pretreatment T: 75 °C P: 550 bar Time: 20 min × 5	Marine	Lipids (18.39 mg/g dw) Fatty acids (17.56 mg/g dw) SFAs (4.74 mg/g dw) MUFAs (5.89 mg/g dw) PUFAs (6.92 mg/g dw) EPA (15.59% dw)	Commercial products	[52]
		Mechanical pretreatment T: 50 °C P: 400 bar Time: 20 min × 5		DHA ^k^ (79.63% dw)		
		Pretreatment T: 40 °C P: 300 bar Time: -		Lipids (34 g/100 g dw) Pigments (38 mg/100 g dw)	Biorefinery	[46]
		Pretreatment T: 40 °C P: 300 bar Time: - Co solvent: 20 wt.% Ethanol		Lipids (45 g/100 g dw) Pigments (100 mg/100 g dw)		
	MAE	T: 100 °C P: - Time: 5 min Power: 800 W Solvent: brine solution		Lipids (16.1% dw)	Food Industry	[133]
	EAE	Pretreatment Enzyme: Snailase + trypsin T: 37 °C pH: 4 Time: 12 h		Lipid recovery (11.73% total lipid)	Biodiesel production	[110]
*Neochloris oleoabundans*	PLE	Pretreatment T: 40 °C P: 1500 psi Time: 20 min Solvent: Limonene	Marine Freshwater	Carotenoids (84.6 mg/g of extract)	Antioxidant activity	[79]
		Pretreatment T: 40 °C P: 1500 psi Time: 20 min Solvent: Ethanol		Carotenoids (120.2 mg/g of extract)		
*Phormidium* sp.	PLE	T: 200 °C P: 20.7 MPa Time: 15 min Solvent: Limonene:ethanol (1:1, *v*/*v*)	Marine	Total Yield (6.8 ± 1.4% *w*/*w*) Lipids (13.0 ± 1.3% *w*/*w*)	Biodiesel production	[83]
*Phaeodactylum tricornutum*	SFE-CO_2_	T: 30 °C P: 30 MPa Time: 60 min Co-solvent: Ethanol 40% (*v*/*v*)	Marine	Fucoxanthin (66.60% *w*/*w*)	Algae biorefinery processes	[143]
		Pretreatment T: 90 °C P: 620 atm Time: -		Lipids (25% dw)	Biorefinery process	[135]
	MAE	T: 170 °C P: - Time: 20 min Power: - Solvent: Ethanol/water (1:2) Magnetic stirred: 1000 rpm		Total yield (48.54% dw) Total phenolic content (56.50 mg GAE/g extract) TEAC (0.529 mmol Trolox eq/g extract) Total carotenoids (1.07 mg/g extract)	Pharmacology sector	[144]
	PLE	T: 110 °C P: 100 bar Time: 20 min Solvent: Ethanol/water (1:2)		Total yield (57.84% dw) Total phenolic content (20.31 mg GAE/g extract) TEAC total antioxidant activity (0.16 mmol Trolox eq/g extract) Total carotenoids (12.51 mg/g extract)		
		T: 40 °C P: 103.4 bar Time: 15 min Solvent: DMSO (100%) Magnetic stirring		Lutein (3.31 mg/L) ß-carotene (2.84 mg/) Fucoxanthin (33.12 mg/L) Diatoxanthin (2.28 mg/L)	Food Industry	[77]
*Porphyridium purpureum*	MAE	T: 40 °C P: 101.325 Pa Time: 10 s Magnetic stirring: 600 rpm	Marine	Phycoerythrin (73.7 ± 2.3 μg/mg per extract)	Cosmetics, Nutraceuticals and used as chemotaxonomic markers	[63]
	UAE	T: room temperature Time: 30 min Power: 70% Solvent: 70% Ethanol/hexane 2:1 (*v*/*v*)		Carotenoids (947.25 µg/g dw) Lipids (21.59 mg/g dw)	Pharmaceutical and Food Industries	[145]
*Scenedesmus dimorphus*	EAE	Pretreatment Enzyme: Snailase + trypsin T: 37 °C pH: 4 Time: 12 h	Freshwater	Lipid recovery (46.81% total lipid)	Biodiesel production	[110]
*Scenedesmus* sp.	SFE-CO_2_	Pretreatment T: 53 °C P: 500 bar Time: -	Freshwater	Lipids (7.41% dw)	Biodiesel production	[146]
	MAE	T: 100 °C P: - Time: 10 min Power: 1000 W Solvent: Chloroform/ethanol (1:1, *v*/*v*)		Lipids yield (% dw) with different drying techniques Freez-drying: 29.65 ± 1.05% Oven drying: 28.63 ± 0.42% Sun drying: 28.33 ± 1.37%	Biodiesel production	[147]
	UAE	T: - Time: 2 min Power: 100 W Solvent: Chloroform/ethanol (1:1, *v*/*v*)		Lipid yield (% dw) with different drying techniques Freez-drying: 19.85 ± 0.35% Oven drying: 18.8 ± 0.1% Sun drying: 18.9 ± 0.5%		
	EAE	Enzyme: Cellulase + pectinase + hemicellulase Concentration: 1:1:1/1:2:1 T: 30 °C/50 °C pH: 3.5/4.5 Time: 72 h		Oil Yield (86.1% dw)	Oil extraction	[113]

^a^ % *w*/*w*: g of dry extract/100 g of dry material; ^b^ dw: dry weight referring to dry algae biomass; ^c^ TEAC: Trolox equivalent antioxidant capacity, antioxidant activity; ^d^ FAMEs: Fatty Acids Methyl Esters; ^e^ MBC: Minimum Bactericidal Concentration, antimicrobial activity; ^f^ MFC: Minimum Fungicidal Concentration, antimicrobial activity; ^g^ SFAs: saturated fatty acids; ^h^ MUFAs: monounsaturated fatty acids; ^i^ EPA: eicosapentaenoic acid; ^j^ PUFAs: polyunsaturated fatty acids; ^k^ DHA: docosahexaenoic Acid.

## Data Availability

Not applicable.
